# p53 mRNA Metabolism Links with the DNA Damage Response

**DOI:** 10.3390/genes12091446

**Published:** 2021-09-20

**Authors:** Sivakumar Vadivel Gnanasundram, Ondrej Bonczek, Lixiao Wang, Sa Chen, Robin Fahraeus

**Affiliations:** 1 Department of Medical Biosciences, Umeå University, 901-87 Umeå, Sweden; ondrej.bonczek@mou.cz (O.B.); lixiao.wang@umu.se (L.W.); sa.chen@umu.se (S.C.); 2 RECAMO, Masaryk Memorial Cancer Institute, Zluty Kopec 7, 656-53 Brno, Czech Republic; 3 Inserm UMRS1131, Institut de Genetique Moleculaire, Universite Paris 7, Hopital St Louis, F-75010 Paris, France; 4 International Centre for Cancer Vaccine Science, University of Gdansk, 80-822 Gdansk, Poland

**Keywords:** DNA damage response, RNA metabolism, p53, MDM2, ATM kinase, RNA-binding proteins, mRNA translation

## Abstract

Human cells are subjected to continuous challenges by different genotoxic stress attacks. DNA damage leads to erroneous mutations, which can alter the function of oncogenes or tumor suppressors, resulting in cancer development. To circumvent this, cells activate the DNA damage response (DDR), which mainly involves cell cycle regulation and DNA repair processes. The tumor suppressor p53 plays a pivotal role in the DDR by halting the cell cycle and facilitating the DNA repair processes. Various pathways and factors participating in the detection and repair of DNA have been described, including scores of RNA-binding proteins (RBPs) and RNAs. It has become increasingly clear that p53’s role is multitasking, and p53 mRNA regulation plays a prominent part in the DDR. This review is aimed at covering the p53 RNA metabolism linked to the DDR and highlights the recent findings.

## 1. Introduction

DNA Damage Processes and Repair Mechanisms

The maintenance of genome integrity is essential for the precise transfer of genetic information. This is being constantly challenged by a range of insults to the cell such as intrinsic (error in replication and reactive oxygen species) or extrinsic cues (chemicals, UV light, ionizing radiations and chemotherapeutic drugs), leading to DNA damage [1,2]. All of these extrinsic and intrinsic attacks lead to the generation of DNA lesions, which include modified bases, mismatches, strand breaks and the crosslink of strands [3,4]. To revert the DNA damage, mammalian cells have developed a sophisticated network of molecular signaling pathways collectively described as the DNA damage response (DDR); each of these pathways repairs a specific type of DNA damage (reviewed in Reference [5]). Briefly, DDR is a multistep phosphorylation-driven signaling cascade, which coordinates the damage recognition, DNA repair processes and cell cycle regulation to ensure the genome integrity. The components involved in DDR are grouped as damage sensors, damage transducers and damage effectors. The damage sensors are activated and recruited to the damage site based on the nature of the damage. Double-stranded breaks (DSBs) are sensed by the MRE11/RAD50/NBS1 (MRN) complex, which recruits and activates ataxia–telangiectasia-mutated (ATM) kinase, which further transduces and activates the damage effectors by a series of phosphorylation events. Besides this, the ATM kinase interacts with Nibrin (NBS1) and phosphorylates H2A histone family member X (γ-H2AX) at serine 139; phosphorylated γ-H2AX functions as a damage sensor and activates transducers [6]. Single-stranded breaks (ssDNA damage) are sensed by replication protein A (RPA) and the RAD9/RAD1/HUS1 complex and activates the Rad3-related (ATR) pathway.

**Figure 1:**
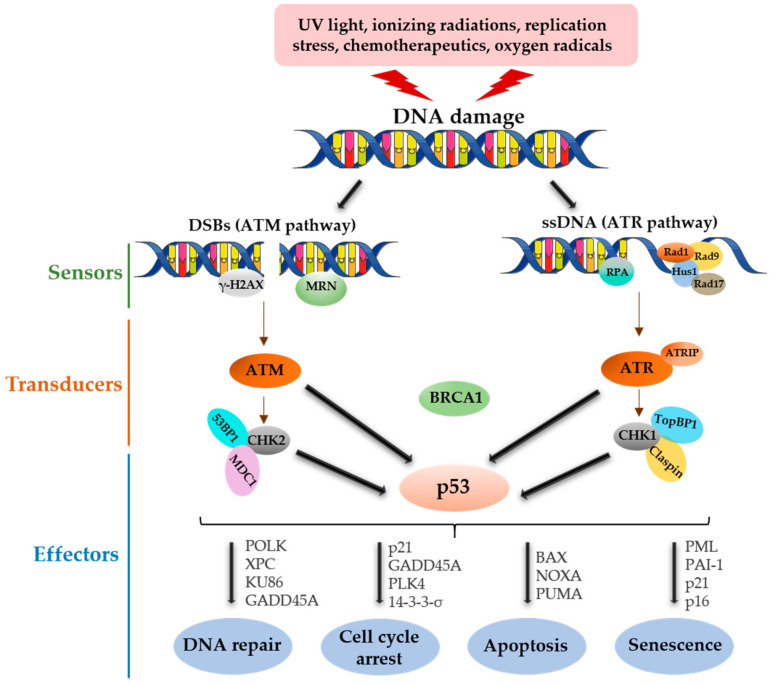
Overview of the DDR and p53 functional roles. Upon DNA damage, the damage sensor proteins sense the DNA lesions, recruit and activate the cascade of transducers, which, in turn, activate the DNA damage effectors that exert an appropriate response to fix the damage and maintain the genome integrity. Double-stranded DNA breaks (DSBs) are sensed by the MRN complex and fixed by the ATM/DNA-PK kinase-mediated response pathway. Single-stranded breaks (ssDNA) are sensed by the RPA and RAD complex, which activates the ATR kinase-mediated response. BRCA1—Breast cancer type 1 susceptibility protein, MDC1—Mediator of DNA damage checkpoint protein 1, 53BP1—p53-binding protein 1, TopBP1—DNA Topoisomerase II-Binding Protein 1, ATRIP—ATR interacting protein, POLK—DNA Polymerase Kappa, XPC—DNA repair protein complementing XP-C cells, GADD45A—Growth Arrest and DNA Damage Inducible α, PLK4—Polo-Like Kinase 4, BAX—Bcl-2-associated X protein, NOXA—Phorbol-12-myristate-13-acetate-induced protein 1, PUMA—p53 upregulated modulator of apoptosis, PML—Promyelocytic leukemia protein and PAI-1—Plasminogen activator inhibitor-1.

Three protein kinases, DNA-dependent protein kinase (DNA-PK), ATM and ATR, are members of the phosphoinositide 3 kinase-related protein kinases (PI3KKs) family, sharing a similar functional domain organization. The ATM kinase is mainly involved in DSBs. The ATR kinase is involved in a broad spectrum of DDR, such as ssDNA damage, DSBs and a variety of DNA lesions generated during DNA replication; thus, the ATR kinase is essential for cell survival. DNA-PK involved in DSBs and in multiple DNA repair pathways, apart from DDR DNA-PK also functions in cell cycle progression, telomere maintenance and transcription regulation. Upon activation, these kinases phosphorylate a series of overlapping substrate pools to facilitate DNA repair and coordinate cell cycle regulation. The ATM and ATR kinases serve as the key signaling factors in DDR; two of the best-researched targets of the ATM and ATR kinases are checkpoint kinase 2 (CHK2) and checkpoint kinase 1 (CHK1), which function together with the ATM/ATR kinases in activating and regulating the effectors. The DDR effectors are substrates of the DDR kinases that, upon activation, participate in a range of cellular processes such as DNA repair, cell cycle control, apoptosis and senescence to ensure the genome integrity (Figure 1) (reviewed in [7,8,9,10]).

Depending on the extent of damage, cells respond either by arresting the progression of the cell cycle and allowing the DNA repair process to fix the damage or evoke cellular senescence or apoptosis if the damage is too severe [11,12]. The DDR is composed of a repertoire of DNA repair pathways, each of which acts based on the nature of damage inflicted. For example, DNA double-stranded breaks are repaired via either homologous recombination (HR) or by nonhomologous end-joining (NHEJ). Mismatches induced by replication errors are corrected by mismatch repair (MMR), small DNA lesions are repaired by base-excision repair (BER) and large nucleotide adducts are corrected by the nucleotide excision repair (NER) pathway. These pathways are not fixed per se; some of the proteins often participate in multiple DDR pathways (reviewed in References [4,11,13,14,15,16,17]).

The failure or erroneous repair of DNA lesions leads to mutations, aberrant gene expressions and chromosomal rearrangements, which pave the way for multiple diseases such as neurodegenerative disorders, aging, immune deficiencies and several types of cancers. Indeed, the prevalence of genomic instability and mutations are still the hallmark of many types of cancers, and the genes controlling DDR components are highly mutated and dysregulated in cancers [11,18].

## 2. p53 Functional Roles in DNA Damage

The p53 tumor suppressor protein was first discovered as a host protein bound to Simian virus 40 large T antigen (SV40) in virus-transformed cells [19,20]. *TP53* is the most frequently mutated gene in many cancers, and its mutations are highly correlated with the poor prognosis of cancer patients [21,22]. p53 is functionally activated in response to several cellular stress stimuli, such as DNA damage, unfolded protein response (UPR), nutrient deficiency and hypoxia [23,24,25,26]. Though vastly regarded as a tumor suppressor protein, p53 also confers pro-survival and oncogenic activities via gain-of-function mutations [27]. p53 is considered as a ‘guardian of the genome’ and orchestrates a variety of DDR pathways and forms the critical link between the DNA damage responses and tumor suppression[18,28,29]. Numerous studies have demonstrated p53-dependent cell cycle arrest and apoptosis in response to DNA damage. Several cellular, as well as animal, models lacking functionally active p53 showed defects in response to DNA damage and tumor suppression (reviewed in References [30,31]). For example, mice lacking p53 are highly prone to cancer, and patients suffering from Li-Fraumeni syndrome, due to germline *TP53* mutations, are at high risk for the early development of multiple tumors [12,32]. The identification of p53′s role in DNA damage dates back to the early 1990s, where multiple studies showed the upregulation of p53 protein levels by DNA damage-inducing agents (ultraviolet light (UV), ionizing radiations and chemotherapeutic agents) [18,33,34,35,36]. Subsequent studies showed that most cellular stress stimuli lead to p53 activation. In addition to its upregulation, p53 is also modified upon DNA damage—in particular, the phosphorylation of serine 15 by the ATM and ATR kinases, a well-established readout of p53 activation during DNA damage (reviewed in Reference [7]). Additionally, DNA-PK phosphorylates p53 at serine 15 and serine 37 residues and activates it by preventing the binding of MDM2 [37]. Following activation, p53 promotes cell cycle arrest at the G1 checkpoint by transcriptionally activating several cell cycle regulatory factors such as the p21 cyclin-dependent kinase inhibitor, thereby facilitating DNA repair and continuing cell division [38,39]. In some cases, p53 also promotes irreversible senescence or apoptosis mediated by several proapoptotic factors, including B-cell lymphoma 2 (Bcl-2), following DNA damage [35,40,41]. So far, it is not well-understood why p53 causes cell cycle arrest or apoptosis, and several factors and contexts contributing towards this discrepancy are detailed in the review by Kastenhuber and Lowe [22]. Furthermore, p53 has essential roles in several DNA repair processes, such as nucleotide excision repair and base excision repair, by activating/regulating the target genes of DNA repair machineries. Both transcription-dependent and -independent functions of p53 are accounted to regulate DNA repair pathways. The diverse functional roles of p53 in each DNA repair process are reviewed in detail by Williams and Schumacher [3].

## 3. p53 mRNA Links with DNA Damage

The differentiation of p53 functional activity responding to each kind of stress response is tightly governed by a hierarchical post-translational modification pattern. Due to the intrinsic disorder nature of p53, these modifications confer allosteric changes in p53 that subsequently differentiate the p53 interactome with myriad cellular factors, thereby determining the physiological outcome of p53 activation [7,42]. Interestingly, the p53 mRNA also plays a vital role in regulating the diverse functional activities of p53.

mRNA translation is highly regulated and interconnected with various cell signaling pathways. mRNAs encode information both in form of sequence, as well as in ordered structures, which are regulated by transacting factors such as translation factors, RNA-binding proteins, ncRNAs and miRNAs which determine the corresponding mRNA translation efficiency. The structural elements present in the untranslated regions (UTRs) of various mRNAs are well-established to play a role in translation regulation. Interestingly, the coding sequences of mRNAs are also reported to regulate translation [43,44,45,46,47,48,49], and the structural elements of such mRNAs were also characterized. The p53 mRNA regulates p53 functional activity via different ways in response to stress signaling pathways, including sequences in the UTRs, as well as the coding sequence. Here, we will focus on the *p53* mRNA regulation linked with the DDR (Table 1), for a general overview of p53 mRNA translation regulation in different cellular stress conditions see the review by Reference [26].

The stress-dependent regulation of p53 translation was first reported from the studies of Kastan et al. where they showed an increase in the new synthesis of p53 proteins following DNA damage without a corresponding increase in the mRNA levels. Subsequent studies demonstrated that more p53 mRNA was associated with polysomes following DNA damage [34].

### 3.1. Regulatory Roles of p53 Untranslated Regions (UTRs) during DDR

UTRs play an important role in regulating the mRNA stability, trafficking, translation efficiency and degradation of the mRNA. Numerous reports have demonstrated the role of these elements in the stress-dependent regulation of p53 mRNA translation (detailed in reviews by References [26,50]).

#### 3.1.1. Roles of 5′ UTR

The 5′ UTR of p53 constitutes about 140 nucleotides and plays an essential role in the stress-dependent regulation of p53 mRNA translation. Early reports showed that the p53 protein could selectively bind to its own 5′ UTR, and this was suggested to suppress its own translation [51]. Takagi et al. showed that ribosomal protein L26 (Rpl26) and nucleolin compete for the binding to the 5′ UTR and regulate *p53* mRNA translation under DNA damage conditions. Rpl 26 binding induces p53 translation, whereas nucleolin leads to a suppression [52]. Subsequently, dsRNA linking the 5′ and 3′ UTR is essential for a Rpl 26-mediated p53 translation increase [53,54]. However, long-range RNA–RNA interactions between the 5′ and 3′ UTR of p53 have yet to be confirmed experimentally. In 2002, Yin et al. showed that Mouse double minute 2 homolog (MDM2) induces translation of the *p53* mRNA from two alternative initiation sites, giving full-length p53 and the p53/47 isoform [55]. Followed by this observation, alternative modes of translation from the internal initiation codon were attributed to the synthesis of the p53/p47 isoform [56,57,58]. In 2006, multiple groups independently reported the internal ribosome entry site (IRES) present in the p53 5′ UTR [59,60,61]. Yang et al. showed that, under DNA damage, the p53 levels are increased by the translation mediated by the IRES element present in the 5′ UTR [61]. Programmed cell death protein 4 (Pdcd4) was reported to bind 5′ UTR and reduce p53 translation under normal conditions; however, during DNA damage, the Pdcd4 levels are reduced, and the suppression of p53 translation is abrogated [62]. Zhang et al. identified the RNA-binding protein RNPC1 (also called RBM38) as a negative regulator of p53 both under normal and stress conditions (including DNA damage); they showed that RNPC1 binds to both 5′ and 3′ UTR of p53 mRNA [63]. In 2014, Death-associated protein 5 (DAP5) (translation initiation factor member of the eIF4G protein family) was identified as a regulatory factor for p53 IRES-mediated translation under different stress conditions, including DNA damage [64].

Works from Halaby et al. showed that the Translation control protein (TCP80) binds p53 5′UTR and is also associated with RNA helicase A (RHA) during DNA damage, having a stimulatory effect on p53 IRES-mediated translation [65]. Ku (Ku70/Ku80) is a highly conserved DNA-binding protein that plays an important role in the DSB repair process. It serves as a DNA damage sensor, interacts with a high affinity to dsDNA ends and with several DNA repair processing factors to provide a scaffold for the repair complex. Under normal conditions, Ku interacts with stem loop structures in the p53 5‘ UTR to suppress p53 translation. During DNA damage conditions, Ku is post-translationally modified by acetylation which abrogates this interaction and relieves the translation suppression [66]. Hence, TCP80 and Ku binding to p53 5′ UTR have opposite effects on p53 expression. Other DNA-damaging agents such as cisplatin and actinomycin-D were reported to stimulate p53 translation, and it was speculated that they cause changes in the phosphorylation status of the RNA-binding protein, heterogeneous nuclear ribonucleoprotein (hnRNP) C1/C2, thereby increasing the binding to the p53 5′ UTR [67]. Another RNA-binding protein hnRNP L was reported to interact with the 5′ UTR and act as a positive regulator of p53 IRES translation during DNA damage [68]. The DNA-damaging agent Etoposide has been reported to stimulate the translation of mouse p53 protein via the interaction of the hnRNP Q protein with the 5‘ UTR of the p53 mRNA (Table 1) [69].

#### 3.1.2. Roles of p53 3′ UTR

3′ UTRs play an essential role in regulating mRNA stability, trafficking and translation. For most of the eukaryotic mRNAs, 3′ UTR mainly contains the poly-A tail. Apart from the poly-A tail, they possess several cis-acting elements such as the AU-rich element, CU-rich element and GU-rich element, which serves as target sites for several transacting regulatory factors. The regulatory role of 3′ UTR in p53 translation during DNA damage emerged from the works of Fu and Benchimol. Using chimeric reporter assays, they showed a negative regulatory role of p53 translation by 3′ UTR, which was relieved upon DNA damage [70]. Later, they identified a cis-acting element (66-nucleotide U-rich sequence) in p53 3′ UTR and its binding partner (unknown 40-KDa protein) responsible for p53 translation repression [71].

After this, several regulatory elements were identified in the 3′ UTR, including AU-rich, U rich and cytoplasmic polyadenylation signals. Works from Mazan-Mamczarz et al. identified the AU-rich-binding protein HuR as an interacting partner of *p53* 3′ UTR, and its binding enhances p53 translation following DNA damage [72]. Interestingly, noncoding RNAs and microRNAs (miRNAs/miR) were reported to compete with HuR for binding to p53 3′ UTR and regulate the translation [73]. Ahuja et al. showed the interplay between HuR and miR-125b in regulating the p53 mRNA translation during DNA damage; the binding of HuR to p53 3′ UTR prevents the miR-125b-mediated translation suppression of p53 [74]. HuR is known to shuttle to the cytoplasm under stress conditions, but it is not well-understood whether this regulates p53 translation during DNA damage. A similar interplay was later reported between miR-1285 and PTB in binding to the p53 3′ UTR under normal and DNA damage conditions [75]. Apart from these, Devany et al. identified a unique feedback loop between p53 and the poly(A)-specific ribonuclease (PARN); under normal conditions, PARN keep the p53 levels low by acting on AU-rich elements at 3′ UTR and destabilizes the p53 RNA levels. Under DNA damage conditions, an increase in p53 activates PARN deadenylase and regulates gene expressions during DDR [76].

Diaz-Munoz et al. showed that the cytoplasmic stress granule-associated RNA binding protein (Tia1) binds to the U-rich element in p53 3′ UTR and mediates p53 translation upon DNA damage induction in B-lymphocytes [80]. Furthermore, works from the Vagner group identified the G-quadruplex RNA structure in the p53 3′ UTR downstream of the poly-A signal. This element was reported to play a role in pre-mRNA 3′ end processing during DNA damage conditions in coordination with RNA-binding protein hnRNP H/F and RNA helicase DHX36 [82,83]. Recently, Lin et al. showed that miR-10b modulates the expression of p53 during the cisplatin treatment by acting on the 3′ UTR and regulates mRNA stability (Table 1) [81].

Intriguingly, a very recent work from Mitschka and Mayr showed that the endogenous expression of both human and mouse p53 was not reliant on its 3′ UTR. They used CRISPR-Cas9 to delete the human and mouse *TP53/Trp53* 3′ UTRs and showed that the endogenous p53 levels were not affected in normal, as well as in DNA damage, conditions. However, when used in isolation, 3′ UTR regulates the gene expression in reporter assays, and this effect was indeed abrogated upon the addition of the p53-coding sequence [84]. This observation again emphasizes the regulatory roles of the p53-coding sequence in p53 expression and activation.

### 3.2. Regulatory Role of the p53-Coding Sequence during DNA Damage

The best-established pathway for p53 activation is the double-stranded DNA damage response pathway (dsDDR). Under normal conditions, the ATM kinase exists as a noncovalently linked inactive dimer. Following activation, the ATM dimer dissociates into active monomers by autophosphorylation at serine 1981 and acetylation at the C-terminal regions. ATM controls p53 activity by phosphorylation at serine 15 (S15), as well as its regulatory partners E3 ubiquitin ligase MDM2 and its nonredundant homolog murine double minute X (MDMX/MDM4). During normal conditions, the N-terminus of MDM2 binds to the Box-I domain of p53 and promotes the polyubiquitination and degradation of p53 [85,86]. However, under DNA damage, ATM phosphorylates MDM2 at serine 395 and switches its activity from a negative to positive regulator of p53 by increasing p53 protein synthesis [44,87]. Further studies showed that an increase in p53 mRNA translation was mediated via the interaction between the C-terminal RING domain of MDM2 and box-1 p53 mRNA sequence. The phosphorylation of MDM2 at serine 395 facilitates the opening of the RNA-binding pocket to bind the RNA structure within +1 to +120 of the p53 coding sequence [78]. Interestingly, it was demonstrated that this interaction also depends on the folding status of the p53 mRNA, which is indeed regulated by ATM-mediated phosphorylation of the MDM2 homolog, MDMX at serine 403. Therefore, MDMX acts as an RNA chaperone to form a platform on p53 mRNA to which MDM2 can bind. This platform consists of three stem loop RNA structures (SL-I, II and III). The central hairpin (SL-II) is the highly conserved box-1, which, under normal conditions, binds to SL-I via interacting with codons 10 and 21. The presence of MDMX switches the interaction of SL-II with SL-III by interacting via codon 22 and codon 41 of SL-III to form the binding platform for MDM2 [79]. MDM2 binding to p53 RNA does not affect the ubiquitination of MDMX under DNA damage conditions, resulting in reduced levels of MDMX (Table 1) [88,89].

The interaction between the box-1 *p53* mRNA and MDM2 is also present in pre-vertebrate *Ciona intestinalis*, where the box-1 RNA stem loop structure facilitates the temperature-based (18 °C) binding to the MDM2 protein [90]. Hence, the folding of the p53 mRNA structure has evolved from temperature regulation in pre-vertebrates to ATM-dependent signaling in mammalian cells; interestingly, MDMX was not present in the pre-vertebrate. This shows that the interplay between MDM2 and p53 is well-conserved during the evolution, while the p53 RNA and MDM2 protein interaction is detected in pre-vertebrates, and the protein–protein interaction evolved in vertebrates (reviewed in Reference [91]). The concept of box-1 stem loop structures of p53 RNA also lies in parallel to the prokaryotic riboswitches, which allows prokaryotes to benefit from a rapid response to changes in the cellular environment by switching the nascent RNA structures. It remains to be seen whether the mammalian system has a similar concept, with only very few examples available thus far. Apart from MDM2 and MDMX, the poly-pyrimidine tract-binding protein (PTB) was reported to interact with the coding sequence, as well as with 5′ UTR of p53 mRNA, and enhance the IRES-mediated translation of p53 isoforms following DNA damage [77].

### 3.3. Synonymous Mutations Regulating p53 Activation during DDR

**Figure 2:**
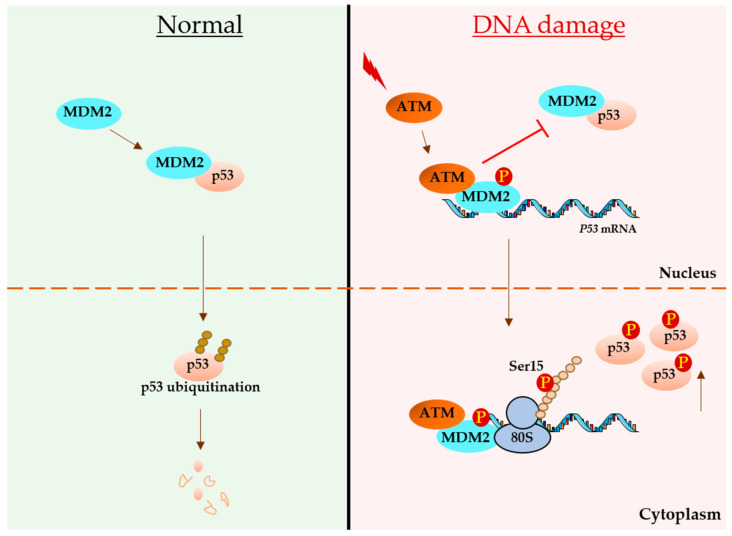
MDM2 switches p53 regulation during DNA damage. Under normal conditions, the MDM2 protein interacts with the p53 protein in the nucleus and tags it for ubiquitination and proteasomal degradation, thereby keeping the p53 levels at minimum. Upon DNA damage, this negative regulation switches to the positive mode. The ATM kinase phosphorylates MDM2 at serine 395, and this allows MDM2 to interact with p53 mRNA and stimulate its translation. MDM2 also brings ATM to p53 polysomes and phosphorylates the nascent p53 peptide at serine 15, and this phosphorylation prevents MDM2-mediated p53 protein degradation.

Synonymous mutations (SMs) are types of point mutations that change DNA/RNA sequences without changing the amino acid sequence. For many years, these mutations are considered neutral and not given enough focus. However, the role of SMs is increasingly emerging associated with cancers and other diseases. The cancer-derived SM CUA to CUG (codon leucine 22), which is located in the box-1 region, affects the folding status of the box-1 stem loop and interferes with the affinity of MDM2 binding, thereby affecting p53 synthesis during DNA damage. This mutation is associated with chronic lymphocytic leukemia (CLL). Other cancer-derived SMs at codon 10 and codon 36 was shown to alter the 5′ terminal region of the p53 coding sequence and affect the interaction with MDM2 [44]. These mutations were shown to reduce p53 synthesis and hinder its activation under DNA damage conditions [44,78]. Interestingly, SMs introduced at codons 17, 18 and 19 (*p53^TriM^*) result in increased p53 RNA–MDM2 interactions even under normal conditions, and this enhances the rate of p53 protein synthesis, as well as degradation, in the MDM2-dependent mode [44]. These observations suggest that the MDM2-mediated synthesis of p53 allows MDM2 to access the nascent p53 protein under normal, as well as in DNA damage, conditions. However, ATM activation during DNA damage leads to increased p53 translation and reduced p53 degradation. A plausible explanation for this comes from a recent study, which showed that MDM2 brings the ATM kinase to p53 polysomes to facilitate the phosphorylation of nascent p53, and this also prevents the binding of MDM2 to the p53 protein, reducing the degradation of the p53 protein [92]. This model provides an example for how mRNA can control the function of its encoded protein (Figure 2).

The phosphorylations at codon serine 15 and serine 20 of p53 play an essential role in regulating p53 functional activities. Interestingly, silent mutations introduced at both these codons (Ser15 > Ser (c.180T > C) and Ser20Ser (c.195A > G)) showed similar influence in p53 activity as that with amino acid changes (Ser15 > Ala, Ser20 > Ala). These observations indicate that nucleotide changes, apart from amino acid changes, also govern p53 activities [44].

## 4. Overview of RBPs Linked with the DDR and Functional Interplay with p53

Recent advancements with large-scale genomic and proteomic studies have led to the discovery of numerous RBPs as key players in the DDR. The functional roles of RNA and RBPs in DDR and DNA repair processes are increasingly emerging, with numerous reports suggesting DDR processes are linked with post-transcriptional gene regulation [93,94,95]. RBPs, together with several ncRNAs, are envisaged to play diverse roles in the DDR. Several RBPs are reported to act directly at the site of DNA damage and facilitate DNA repair processes by interplaying with multiple DNA repair proteins and, also, with p53 (Table 2 provides the brief overview of RBPs associated with the DDR and their functional interplay with p53). For example, hnRNP C and hnRNP UL 1 and 2 are binding partners of DNA repair complexes BRCA1/BRCA2/PALB2 and MRN, respectively, and play essential roles in promoting ATR-dependent signaling and HR-mediated repair [93,94]. RNA-binding motif protein (RBM) X binds to dsDNA breaks and protects them from degradation, thereby increasing the fidelity of the repair [94]. FUS and NONO are recruited to dsDNA break sites in a PAR-dependent manner and are involved in NHEJ and HR. Prp19 is a ubiquitin ligase involved mainly in splicing and is reported to localize the DNA damage site via binding to RPA and facilitate the ATR response [93]. Recently, RBM14 was reported to interact with Ku80 and participate in the NHEJ DNA repair pathway. Interestingly, RNA binding does not account for the recruitment of several RBPs to the DNA damage site, suggesting that these RBPs are recruited either by interacting with the DNA template or via DNA repair proteins [94].

Apart from the direct roles in DDR, RBPs also play a key role in regulating the gene expression of DDR and checkpoint proteins at both transcriptional and post-transcriptional processes (reviewed in References [94,95,129]). RBPs also bind to an increasing number of long noncoding RNAs (lncRNAs) and, together, orchestrate transcription and chromatin remodeling processes [94]. The role of multiple RBPs in regulating p53 expression during DNA damage was already discussed above. Additionally, many RBPs have been reported to play a key role in preventing DNA damage by controlling R-loop (three stranded nucleic acid structures consist of a DNA–RNA hybrid and unpaired single-stranded DNA) formation and DNA breaks [130].

Consequently, using functional screens and proteomic studies, DNA damage-signaling proteins are identified to post-translationally modify several RNA-processing factors, including RBPs [94]. Many RBPs are directly phosphorylated by DDR signaling kinases such as ATM, ATR, DNA-PK (sensors) CHK1 and CHK2 (transducers); this regulates the RBP activities in DNA damage. Apart from phosphorylation, RBPs are also modified by poly (ADP-ribosylation) or parylations and acetylations. DNA damage-signaling pathways are also reported to highly modulate the subcellular localization and abundance of many RBPs during DNA damage (reviewed in Reference [94]).

## 5. Conclusions and Perspectives

Despite being intensively researched for many years, p53 still remains a central candidate of interest in the cancer research field with its ever-emerging functional roles in multiple cellular processes. There are still many questions left unanswered about its regulated activity under different cellular stresses. p53 mRNA regulation has become one of the important facets in the functional activation of p53 in response to various cellular stress conditions. In the DDR, p53 mRNA plays a pivotal role in p53 activation. Many RBPs, including transacting translation factors and microRNAs, were identified to bind multiple regions of p53 mRNA and regulate its translation. It is likely that more such factors will be uncovered in the near future. However, it is unclear whether these factors coordinate in regulating p53 activation during DNA damage. If they function together, it remains to be seen how these factors coordinate to orchestrate p53 mRNA translation. So far, the molecular mechanism of some of these factors have been well-established with multiple RNA–protein biochemical techniques and functional studies. The molecular action of other p53 mRNA-binding factors during DNA damage need to be studied in detail.

The ATM kinase activates p53 during DNA damage; it is interesting to note that ATM regulates both the folding of nascent p53 mRNA as well as the encoding nascent peptide. It will be fascinating to know if this represents a broader model of how signaling pathways integrate the synthesis and degradation of a protein. In line with this, it would be interesting to see how signaling pathways regulate individual mRNA translations under certain cellular stress conditions. It will be a likely scenario that the structural rearrangement of p53 mRNA during DNA damage contributes to its translation regulation and the binding of various proteins. Future studies should focus on determining the native p53 RNA structure under different stress conditions and identifying the protein contact points in p53 RNA. Emerging observations indicate a potential linkage and functional dependence between the post-transcriptional modifications of mRNA and their structures; it will be interesting to investigate such modifications in p53 mRNA and their regulatory roles in p53 gene expression.

Recent observation based on CRISPR-Cas9 knockdown have indicated that the endogenous p53 levels are not reliant on regulation by p53 3′ UTR [84]; these results are intriguing and, at the same time, strengthen the regulatory roles of the p53 mRNA coding sequence. Similar studies should be done to access the functional role p53 5′ UTR in regulating the endogenous p53 levels.

## Figures and Tables

**Table 1:** Summary of different p53 mRNA regions involved in DDR.

Binding Factor	Binding Region (p53)	Function	Reference
Nucleolin	5′ UTR	suppresses p53 translation	[52]
Rpl26	5′ UTR	enhances p53 translation	[52]
Pdcd4	5′ UTR	inhibits p53 translation; during DNA damage, the Pdcd4 levels are reduced, and p53 translation suppression is abrogated	[62]
TCP80	5′ UTR (IRES I)	stimulates p53 translation	[65]
Ku÷(Ku70/Ku80)	5′ UTR (IRES I)	represses p53 translation; during÷DNA damage, the Ku protein is modified and abrogates binding to p53 mRNA and inhibits repression	[66]
hnRNP C1/C2	5′ UTR	stimulates p53 translation	[67]
hnRNP L	5′ UTR	stimulates p53 translation	[68]
hnRNP Q	5′ UTR	stimulates p53 translation	[69]
Dap5	5′ UTR (IRES I) and coding sequence (IRES II)	promotes IRES driven translation	[64]
PTB	5′ UTR (IRES I), coding sequence (IRES II) and 3′UTR	regulates p53 translation	[75,77]
RBM38/RNPC1	5′ UTR and/3′ UTR	represses p53 translation	[63]
MDM2	Coding sequence (IRES II)	enhances p53 translation	[44,78]
MDMX÷(MDM4)	Coding sequence (IRES II)	chaperoning p53 mRNA to facilitate MDM2 binding and enhances p53 translation	[79]
HuR	AU-rich element (3′ UTR)	stabilizes p53 mRNA and enhances translation.	[72]
miRNA-125b	AU-rich element (3′ UTR)	competes with HuR for binding to p53 mRNA and suppresses translation	[74]
PARN	AU-rich element (3′ UTR)	destabilizes p53 mRNA and decreases p53 levels under normal conditions	[76]
Tia1	U-rich element (3′ UTR)	targets p53 mRNA to stress granules under normal conditions; during DNA damage, enhances p53 translation	[80]
40 kDa unknown protein	U-rich element (3′ UTR)	negatively regulates p53 levels under normal conditions, which were relieved during DNA damage	[70]
miR-10b	3′ UTR	regulates the stability of p53 mRNA during cisplatin treatment	[81]

**Table 2:** Overview of RNA binding proteins (RBPs) linked with the DDR and functional interplay with p53.

RBP	Main Feature	Functional Interplay with p53	Reference
AGO2	RNA interference	p53 regulates AGO2 association with miRNAs and remodel miRNA-mRNA network during DDR	[96,97,98]
BRCA1	E3 ubiquitin-protein ligase	DNA damage sensor; regulates p53 dependent gene expression	[99,100,101,102]
CCAR2	nuclear protein	activates p53 and induction of p53 dependent apoptosis	[103,104]
DAP5 (EIF4G2)	translation initiation factor	stimulates p53 translation under different stress conditions	[64]
DDX5÷(p68)	ATP-dependent RNA helicase, transcriptional regulator	required for p53 dependent p21 induction and cell cycle arrest	[105,106]
DHX9 (RHA)	ATP-dependent RNA helicase	enhances expression of p53	[65,107]
hnRNP F/H	nuclear ribonucleoprotein	essential for maintaining p53 pre-mRNA 3′-end processing	[83]
hnRNPC 1/2	nuclear ribonucleoprotein	interacts with p53 5′ UTR and stimulates translation	[67]
hnRNPUL-1	transcription regulator	interacts with p53 and inhibits its transcriptional activity during DDR	[108,109]
HuR	RBP; interacts with 3′ UTRs of mRNAs	increases the stability of p53 mRNA and translation	[72,110,111]
Ku70-Ku80÷(XCCR5-XCCR6)	single-stranded DNA-dependent ATP-dependent helicases; DNA damage sensor	interacts with p53 5′ UTR and suppresses translation under normal conditions; during DDR, Ku protein is modified and releases the suppression of p53 translation	[112,113,114]
MDM2	E3 ubiquitin ligase	enhances p53 translation during DDR	[26,78,87,115]
NOP53	nuclear protein	DNA damage sensor, essential for stabilization of p53 protein	[116,117,118]
Nucleolin	nucleolar protein; multifunctional phosphoprotein	represses p53 translation	[53,54]
PCBP4÷(MCG10)	poly(C)-binding protein	p53 activates PCBP4 during DDR and induces apoptosis and cell cycle arrest	[119,120]
PDCD4	apoptosis regulation protein	suppresses p53 in normal condition, under DDR PDCD4 levels are reduced	[62]
RBM38÷(RNPC1)	RBP; regulates mRNA stability	represses MDM2 and p53 translation	[63,121]
RBMX÷(hnRNP G)	nuclear ribonucleoprotein	p53 enhances the expression of RBMX and promotes DNA repair	[122,123]
RPL26	60S ribosomal protein	enhances p53 translation after DNA damage	[52,53,54]
RPS27L	ribosomal protein	direct p53-inducible target, interferes with p53-MDM2 axis	[124,125]
TCP80 (IL3)	regulates p53 IRES translation	interacts with 5′ UTR and stimulates p53 translation	[65,107]
YB-1 and EWS	multifunctional nucleic acid binding proteins	regulate MDM2 splicing and increase p53 levels during DDR	[126,127,128]
